# Supplementary material for: The assignment and distribution of the dyslexia label: Using the UK Millennium Cohort Study to investigate the socio-demographic predictors of the dyslexia label in England and Wales
Source: PLoS One. 2021 Aug 24;16(8):e0256114. doi: 10.1371/journal.pone.0256114 (PMC8384203; doi:10.1371/journal.pone.0256114)
Supplement: Attachment — Submitted filename: PONE-D-21-02074-R1 Response to reviewers.doc [file pone.0256114.s001.doc]

We thank the two reviewers and editor for their useful comments and feedback on our manuscript; the resulting changes have clearly improved our paper and the clarity of its contributions, and we now feel it is ready for publication in PLOS ONE. Please see the table below where we detail how we have responded to the reviewers’ feedback.

| **Reviewer Comment** | **Response** |
| --- | --- |
| Reviewer 1 | |
| Thank you for the opportunity to review this manuscript that reported an interesting and important study. | Thank you for your positive comments and feedback, which have improved our paper and the clarity of its contributions. |
| The title could be further developed to emphasize that the study investigated factors correlated with the application of the diagnostic label – as opposed to ‘dyslexia’ per se. | Agreed; we have amended the paper title to:  *The assignment and distribution of the dyslexia label: Using the UK Millennium Cohort Study to investigate the sociodemographic predictors of the dyslexia label in England and Wales*  To make it explicitly clear that we are referring to the assignment of the dyslexia label. |
| Similarly, the page 3 subheading and text with reference to ‘dyslexia’ and ‘autism’, please consider further clarifying the use of the label as opposed to specific condition (there may be other areas of the manuscript where this may be appropriate too). | Agreed; we have amended the subheadings, as well as related links/references in this section (see page 3 onwards). |
| I also suggest that specific / explicit statement of the research question/s are included in the 'The Present Study' subsection, and for explicit links to these in the discussion. | The research question has been included on page 10, and referred back to on page 20. |
| Thanks for a well-written manuscript. | Thank you for your positive comments and feedback, which have improved our paper and the clarity of its contributions. |
| Reviewer 2 | |
| This is an interesting manuscript that looks at demographic factors associated with having a formal dyslexia diagnosis among 11-year-olds in the UK. I have two major questions/concerns as well as a few more minor suggestions for improvement of the work. | Thank you for this positive feedback. We outline how we address all of these major and minor comments below. |
| My primary concern begins with a question about how the authors frame the ‘debate’ that motivates the manuscript. I disagree with citing only Elliott on this question in the introduction. I think the view now widely held among experts in the field is that the terms “dyslexia,” “word-level reading difficulties,” “specific learning disability in reading,” and several others are interchangeable. In other words, dyslexia represents the low tail of the distribution of basic literacy skills (word reading, decoding, fluency, spelling). Apart from a few exclusionary conditions (e.g., literacy problems due to lack of instruction, sensory disabilities, or intellectual disability) there is not compelling evidence that the etiology, brain bases, neuropsychology, or treatments for “dyslexia” and “low reading achievement” are different. We know unquestionably that a substantial portion of children have difficulties acquiring basic literacy skills in the absence of these exclusionary criteria and that those difficulties are functionally impairing. Thus, to me, the question is not so much whether dyslexia is a valid diagnosis, but how access to accurate diagnosis and relevant resources for individuals with literacy problems varies according to the demographic factors the authors are exploring. | Thank you for this comment — we entirely agree with your point. The present study does not aim to investigate whether dyslexia is a valid diagnosis, but aims to understand how diagnosis varies by socio-demographic factors.  To make this clearer, we have added further literature in the opening discussion (page 3), and have clarified this in the research question (page 10).  While we are unable to directly identify whether “accurate diagnosis and relevant resources for individuals with literacy problems varies according to the demographic factors the authors are exploring”, we agree this wider work would be of interest, and we have included this as an area for potential research in the future (see page 23). |
| Related to this issue, there are multiple points throughout the manuscript where the authors refer to whether individuals “have dyslexia” where I think what they mean is whether the person has been “diagnosed with dyslexia” or “identified as having dyslexia.” (E.g., lines 28, 124, 230, 385/386, 520). This may seem like a minor point, but I think it is important to the framing of the manuscript and the take-home message. For many medical and psychological diagnoses, we know there are disparities across demographic groups in getting an accurate diagnosis as well as access to treatments. Take hypertension as an analogy. Let’s say we looked at a large group of people and found those from higher SES backgrounds were more likely to have a formal diagnosis of hypertension, but that the risk of having objectively measured high blood pressure was actually higher in individuals from a lower SES background. I don’t think we would conclude that hypertension is associated with higher SES, or that those from higher SES are more likely to have “hypertension” while those from lower SES are more likely to have “high blood pressure.” I think we would more likely say that lower SES conveys increased risk for hypertension AND that there is an SES-related disparity in how often accurate diagnosis is made. Also important, this disparity would not cause us to question whether hypertension is a valid or meaningful diagnosis. | Thank you for this comment. We agree, and have thus made a number of changes throughout the manuscript (including all of the ones that have been identified by the reviewer) in order to make it clear that when we are discussing our results, we are talking about those who have been identified with dyslexia (please see manuscript with tracked changes to see where these edits have been made throughout the paper including the title and abstract). |
| A more minor, but related point, is that in the current manuscript, determination of whether a child had been diagnosed with dyslexia was made solely by teacher report. It is possible that teacher awareness of children’s diagnoses (or of the meaning of a dyslexia label) could vary systematically as a function of some of the demographic variables the study is exploring. The authors allude to the limitations of this approach in the methods, but I was surprised that they did not include a limitations section in the discussion reviewing this and other limitations of the work. I also suggest being clearer throughout the manuscript by using language such as “having a teacher-reported dyslexia diagnosis” or similar. | We have included a new limitations section (see page 24) which explicitly highlights the limitations that you raise. We also make it clear in the research question and subsequent discussion that we are referring to teacher identification of dyslexia. |
| My second major concern has to do with how the authors handled IQ (the “Cognitive profile” section on page 14). I did not understand exactly what they did or why. This issue should be set up better in the introduction and probably linked to the questions that motivates the manuscript. The field has moved away from IQ-discrepancy definitions of dyslexia again because of empirical evidence that the underlying causes of, and appropriate treatments for, dyslexia do not depend on IQ score. Therefore, I think it is probably not appropriate for primary analyses to control for verbal and nonverbal abilities. However, given the historic emphasis on IQ discrepancy in defining learning disabilities, it could be appropriate to conduct secondary analyses that control for IQ and see whether the pattern of results changes. If the authors go that route, I suggest explaining the motivation for their approach (if possible, along with a priori hypotheses) in the introduction. | Thank you for this comment, and the opportunity to clarify this further in the manuscript.  As discussed above, the purpose of this paper is not to understand whether the children in the study have a ‘valid’ label of dyslexia; rather it seeks to understand what socio-demographic factors influence the probability of dyslexia being identified by a child’s teacher.  In order to answer this question, it is necessary to control for ability. It is feasible to suggest that identification of dyslexia is directly related to verbal ability (as is demonstrated by the significant relationship shown in Table 4 (p<0.001). Therefore, if this is not controlled for, a significant relationship between the dyslexia label and the independent variables may be due to the confounding relationship between verbal ability and the variable, and not due to the dyslexia label.  Furthermore, non-verbal ability is also controlled for as a confounding variable, as the child's non-verbal ability may also interact with the socio-demographic variables of interest. It may also impact whether a teacher identifies a child as dyslexic (perhaps opposed to another label). Therefore, it is necessary to take this into account in order to isolate the variables of interest.  However, we acknowledge this should be made more explicit in the paper; we have therefore included a new sentence to make this clearer on page 15. |
| Line 69: “child having autism” – better to say “being diagnosed with autism”? | Agreed; this has been fixed (see page 3) |
| Line 185: I think there is a typo in this line | Apologies; this has been fixed (see page 8). |
| Line 252: “educational” – should be “education”? | Apologies, this has been fixed (see page 11) |
| Paragraph beginning on line 440: The Shaywitz and Peterson/Pennington explanations are different versions of the same thing, not competing explanations. One of the reasons that girls tend to demand less attention in the classroom than boys is because they have lower rates of hyperactive and impulsive symptoms. | Agreed; we have amended the references to these two pieces of literature in this paragraph to reflect this coherence (see page 22). |
| Q3. Have the authors made all data underlying the findings in their manuscript fully available? No. | Yes. As per our Data Availability Statement on the original submission: “*The data that support the findings of this study are openly available via the UK Data Service (funded by the ESRC) at: http://doi.org/10.5255/UKDA-SN-5350-5, reference number [5350-5]; http://doi.org/10.5255/UKDA-SN-5795-5, reference number [5795-5]; http://doi.org/10.5255/UKDA-SN-6411-8, reference number [6411-8]; http://doi.org/10.5255/UKDA-SN-7464-5, reference number [7464-5]; http://doi.org/10.5255/UKDA-SN-8156-7, reference number [8156-7].*” (see page 27) |
| General | |
| General formatting, structure, tidying and fixes | Further to the main reviewer comments above, we have made minor tweaks throughout the manuscript to enhance readability, structure and narrative. We have also fixed a number of small typographical errors, as well as clarified consistency of use of terminology throughout.  Furthermore, we have checked and updated the literature throughout the manuscript, including tidying up the formatting of all references, as well as some small typographical errors (see pages 28-32). |
