# Supplementary material for: The assignment and distribution of the dyslexia label: Using the UK Millennium Cohort Study to investigate the socio-demographic predictors of the dyslexia label in England and Wales
Source: PLoS One. 2021 Aug 24;16(8):e0256114. doi: 10.1371/journal.pone.0256114 (PMC8384203; doi:10.1371/journal.pone.0256114)
Supplement: Attachment — Submitted filename: PONE-D-21-02074-R2 table of changes.docx [file pone.0256114.s002.docx]

| **Reviewer comment** | **Response** |
| --- | --- |
| *Journal requirements* | |
| Please review your reference list to ensure that it is complete and correct. If you have cited papers that have been retracted, please include the rationale for doing so in the manuscript text, or remove these references and replace them with relevant current references. Any changes to the reference list should be mentioned in the rebuttal letter that accompanies your revised manuscript. If you need to cite a retracted article, indicate the article’s retracted status in the References list and also include a citation and full reference for the retraction notice. | The reference list has been reviewed and updated with appropriate citation information. No retracted articles have been cited in our paper. |
| *Reviewer 2* | |
| Page 6, second paragraph, line 4: Do you mean “*more boys than girls*” instead of “*more girls than boys*”? | Thank you for identifying this error — this has been corrected. |
| Page 8: The data on the relationship between ethnicity and special education eligibility in the U.S. are complex, and this is a controversial topic. While a higher percentage of ethnic minorities are identified for special education than white students, this is driven largely by inclusion in categories related to behavior. There is actually evidence that minority students are under-represented in special education categories most relevant to learning disabilities (including dyslexia, and so of relevance to the current paper). (Morgan et al., 2015). The authors may want to tweak this section to reflect the complexity of this issue.  *Morgan, P. L., Farkas, G., Hillemeier, M. M., Mattison, R., Maczuga, S., Li, H., & Cook, M. (2015). Minorities are disproportionately underrepresented in special education: Longitudinal evidence across five disability conditions. Educational Researcher, 44(5), 278-292.* | Thank you for making us aware of this work — we have added a reference to this paper (as well as refined this section) to better demonstrate the complexity of the relationship between ethnicity and special educational needs. |
| Page 15: I still find the explanation of what exactly the authors did with IQ and why confusing. Also, this issue is still not addressed in the introduction. Based on what I understand from the paper, here are my suggestions.  First, the authors actually do look at the associations between variables of interest both with and without accounting for IQ (i.e., the bivariate results on page 19 versus the regression results beginning on page 20). To the extent that the pattern of results is similar in both cases, this would strengthen the conclusions they can draw. Perhaps they can highlight this a bit more.  Second, I think the example given on page 15-16 about why it is “necessary” to control for IQ is confusing. I don’t agree that it is necessary, but I agree it provides a stronger test of some of their research questions. The explanation might be clearer if the authors gave an example of how IQ could cause a spurious relationship, rather than no relationship. I think the most likely way in which IQ would confound the results is as follows: Children with higher IQ are more likely to be diagnosed with dyslexia because many clinicians and teachers still embrace an IQ-discrepancy model of dyslexia, which essentially requires an average to above average IQ. Higher IQ could also be associated, on average, with higher social class for a wide variety of reasons. Therefore, it might appear that there is an association between higher social class and the dyslexia label, but this is really an artifact of a reliance on IQ-discrepancy definitions. Therefore, controlling for IQ in the regression analyses provides a stronger test of whether social class and other variables of interest are independently associated with the dyslexia label than the bivariate analyses. | Thank you for this comment. We have added in further explanation of why cognitive profile variables were used and used the example you kindly suggested.  We would also like to reiterate that we were not controlling for ‘IQ’ per se, and that verbal ability was also used as a control variable. We hope that the elaboration provided better explains this in the context of the study and the research question. |
| Page 21—I recommend explaining the direction of all these effects here (e.g., which gender, which direction for age in year group, which direction for SES, education, and income) rather than in the opening of the discussion. | Agreed; we have included an explanation of the direction in this section. |
| Page 22—As mentioned above, I think most of the first paragraph of the discussion section would fit better in results. | Agreed; this has been moved into the Results section and further expanded. |
| Concluding remarks: here and in a few other places throughout the paper I think the language about factors being “*unrelated to dyslexia*” or there being “*no biological reason*” may be too strong. For example, there are a number of possible reasons that factors associated with SES could play a true etiologic role in the development of dyslexia or other learning disabilities, and the current paper cannot address that directly. Similarly, there are potential biological reasons that being born in a certain month could convey risk for particular developmental outcomes (e.g., higher likelihood of exposure to certain prenatal infections). I agree those are not the most likely explanations for the results in this paper, but again, the design cannot address them directly. So it might be better simply to soften the language throughout to say things like “*unlikely to be related*.” I also recommend being careful not to imply that somehow all biological effects are genetic and all environmental effects are not biological. Many environmental effects act directly on biology. | Agreed; the language has been clarified and toned down around these claims as noted to make it clear that relationships between the variables of interest and dyslexia would be unlikely to be to do with biological reasons. |
| *General* | |
| General formatting, structure, tidying and fixes. | Further to the main reviewer comments above, we have made minor tweaks throughout the manuscript to enhance readability, structure and narrative. We have also fixed a number of small typographical errors.  Furthermore, we have checked and updated the literature throughout the manuscript, including tidying up the formatting of all references, as well as some small typographical errors (see pages 28-32). |
